# Vitamin D alleviates neurotoxicity induced by propofol anaesthesia in the offspring of mice

**DOI:** 10.1371/journal.pone.0349784

**Published:** 2026-05-22

**Authors:** Hüseyin Cihat Solmaz, Feride Karacaer, Ebru Biricik, Demet Tunay, Müge Can, Samet Kara, Kübra Akıllıoğlu, Sait Polat

**Affiliations:** 1 Çukurova University, Medicine Faculty, Anesthesiology and Reanimation Department, Adana, Turkiye; 2 Adana Yüreğir State Hospital, Adana, Turkiye; 3 Çukurova University, Medicine Faculty, Department of Histology, Adana, Turkiye; 4 Çukurova University, Medicine Faculty, Department of Physiology, Adana, Turkiye; Zhengzhou University, CHINA

## Abstract

**Background:**

Maternal propofol can induce neurotoxicity in the developing brain. Vitamin D modulates neurotrophin and neuromediator synthesis, reduces neuroinflammation and neuroapoptosis. The present study examined whether vitamin D could mitigate maternal propofol induced neurotoxicity in the offspring.

**Method:**

Twenty pregnant mice were randomly divided into four groups. During the pregnancy, the groups designated as D and PD were given intraperitoneal vitamin D, while the groups labelled as P and C received 1 ml of saline. On the 14th day of pregnancy, groups P and PD received 200 mg kg^-1^ of propofol intraperitoneally. Newborn mice from the aforementioned groups were included in the study. The hippocampus and the prefrontal cortex were examined by immunohistochemistry at two postnatal ages: 7 and 45 days (PN7 and PN45). A battery of behavioural assessments was conducted using open field and elevated plus maze tests to evaluate potential alterations at PN45.

**Results:**

Vitamin D reduced the expression of inflammatory and apoptotic factors induced by propofol, such as TNF-α (group P versus group PD: 0.79 (0.76–0.81) versus 0.36 (0.34–0.39), %95 CI; p < 0.001), Bax (group P versus group PD: 0.72 (0.69–0.74) versus 0.52 (0.5–0.54), %95 CI; p < 0.001) in the hippocampus in PN7. The administration of vitamin D resulted in enhanced expression of anti-apoptotic and neuroprotective proteins, such as Bcl-2 (group P versus group PD: 0.3 (0.27–0.33) versus 0.55 (0.53–0.57), %95 CI; p < 0.001) in the hippocampus in PN7. These changes also occurred in the hippocampus in PN45 and the prefrontal cortex in PN7 and PN45. No significant differences were identified in the behavioural tests between the groups.

**Conclusion:**

Vitamin D treatment during pregnancy could prevent neurotoxicity in the offspring of mice exposed to propofol.

## 1. Introductıon

Experimental and animal studies have shown that the central nervous system (CNS) is highly sensitive to agents used in general anaesthesia during the intrauterine period and early postnatal life [1,2]. As human brain development, including neuronal formation, maturation and apoptosis, begins in the second trimester of pregnancy and continues actively throughout the perinatal period, the human CNS is also vulnerable to anaesthetics [3].

Propofol is a lipophilic intravenous agent that is widely used in the induction and maintenance of general anaesthesia due to its rapid onset and recovery, and low side effect profile [2]. In recent years, studies conducted on animals and in cell culture models have suggested that propofol anaesthesia may cause cognitive impairment and neurotoxicity in the developing brains of neonates and foetuses [4]. Thanks to current medical advances, anaesthetic agents are commonly used in paediatric cases and approximately 1–2% of pregnant women require general anaesthesia for foetal or emergency surgery [5]. Therefore, the possibility that propofol-induced neurotoxicity, as observed in animal models, may also affect to humans must be taken into consideration.

Several mechanisms associated with propofol-induced neurotoxicity have been identified. As many developmental processes, including cell proliferation, neurogenesis, synaptogenesis and neuronal remodelling, occur during the intrauterine and neonatal periods, propofol exposure may result in various outcomes in the developing brain [6]. These outcomes include neuroinflammation, neuroapoptosis, oxidative DNA damage, supression of neurogenesis, impaired dendritic spinogenesis, mitochondrial fission, and epigenetic dysregulation (microRNAs) [6,7]. Exposure to propofol during the early pregnancy impairs neurogenesis and cognitive function in offspring rats by reducing levels of brain-derived neurotrophic factor (BDNF) and synaptophysin through the inhibition of histone acetylation [8,9]. Furthermore, anaesthetic agents that bind to GABA and NMDA receptors in immature neurons induce apoptosis by activating these receptors rather than inhibiting them [10].

Studies conducted on neonatal mice have demonstrated that propofol administered at a dose lower than that used for anaesthesia can induce neuroinflammation, neuroapoptosis and result in a persistent decline in dendritic growth [11]. These data suggest that the immature brain is susceptible to propofol-induced neurotoxicity by inhibiting long-term neuronal potentiation and causing neurobehavioural dysfunction.

Vitamin D (vit D) is a cholesterol-derived hormone that plays a critical role in brain development, in addition to its fundamental role in calcium metabolism. Both neurons and microglia express the vit D receptor (VDR), and vit D is involved in the expression of key enzymes responsible for proliferation, differentiation, neurotransmitter anabolism, neurotrophy, neuroinflammatory regulation and neurotransmission [12]. Vit D has an immunomodulatory effect, inhibiting neuroinflammatory processes in the brain [13]. It contributes to myelination by inducing the differentiation of neural stem cells and oligodendrocytes [14], and it up-regulates neurotrophins such as BDNF [15]. An experimental study has demonstrated that vit D supplementation improves impaired hippocampal neurogenesis and reduces neuronal apoptosis [16].Vit D has been documented to be a neuroactive steroid with neuroprotective properties that improve learning and memory [16].

We hypothesized that vit D could prevent propofol-induced apoptosis and neuroinflammation. Our study aimed to investigate the effects of gestational vit D administration on neurotoxicity in offspring exposed to propofol in the second trimester of pregnancy. The primary outcome of the study was to examine the effect of vit D on propofol-induced neuroinflammation and apoptosis.

## 2. Method

### 2.1. Animals

All experimental procedures were approved by the Ethics Committee of Cukurova University Medical Sciences Experimental Application and Research Center (Adana, TURKEY, protocol number: 05.06.2023–3/5). The study used a randomised and controlled experimental animal model and was conducted between June 2023 and July 2024, using the minimum number of animals necessary. The Animal Research: Reporting In Vivo Experiments (ARRIVE) guidelines were used during this study to ensure transparent, complete, and reproducible reporting of experimental procedures [17]. Animal care, and follow-up practices were carried out in accordance with Institutional Animal Care and Use Committee (IACUC) guidelines during the experiment. Adult Swiss albino mice of breeding age (8 weeks old; 27 ± 2 g; 20 females, 5 males) were provided by the Animal Centre of the Çukurova University Medical Faculty. Female mice mated with male mice in a 2:1 ratio. All mice were housed at a constant temperature (21–23^o^C) and humidity (40%–60%) under a 12 h light/dark cycle and allowed free access to a standard diet and clean drinking water.

### 2.2. Pregnancy timing

**Fig 1:**
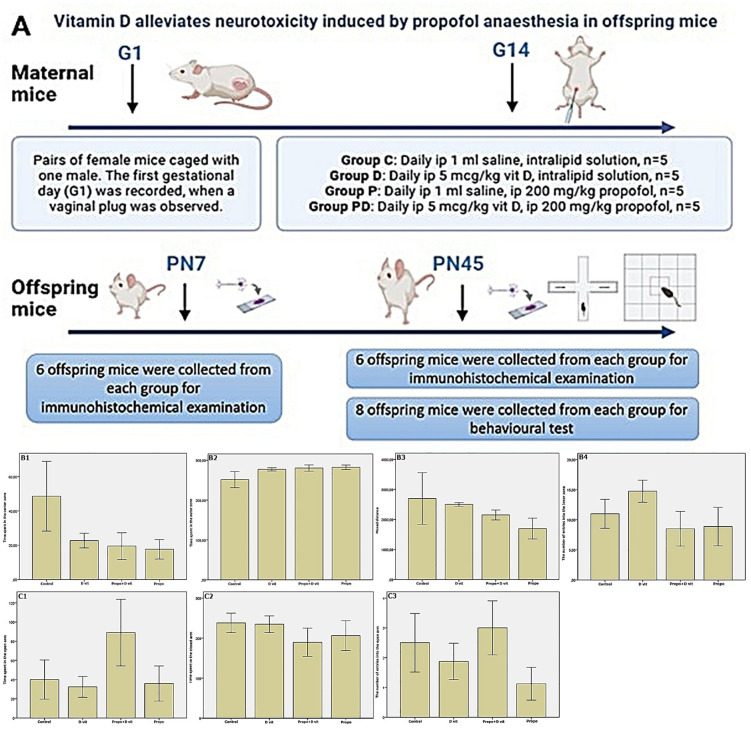
Study design and open field test results. A, Flow diagram of the present study. B, Results of open field tests. B1, time spent in the center zone; B2, time spent in the outer zone [H(3,32) = 2.18, p = 0.53; figure 1-B1, figure 1-B2]. B3, the distance travelled [U(3,32) = 4.47, p = 0.21; figure 1-B3]. B4, the number of entries into the center zone [U(3,32) = 4.39, p = 0.22; figure 1-B4]. Kruskal Wallis followed by Mann Whitney U test. (n = 8). C, Results of elevated plus maze tests. C1, time spent in the open arms [H(3,32) = 2.25, p = 0.52; figure 1-C1]. C2, time spent in the closed arms [H(3,32) = 1.23, p = 0.74; figure 1-C2]. C3, frequency of entering the open arm [H(3,32) = 2.90, p = 0. 40; figure 1-C3]. Kruskal Wallis followed by Mann Whitney U test (n = 8 in each group)‌‌.

Two female mice were mated with one male mouse overnight for breeding purposes. The day on which the presence of a vaginal plug was observed was designated the first day of pregnancy (G1). At the 14th day of pregnancy (G14), pregnant mice were housed separately. The offspring born to these mice were utilised in this study (Fig 1A).‌‌

Care for the enrolled offspring was conducted under the guidance of the ARRIVE and IACUC protocols, which included daily health and behavior checks. In accordance with the study methodology, offspring were anesthetized with sodium pentobarbital (50 mg kg^-1^) and sacrificed via cervical dislocation by trained personnel at postnatal day 7 (PN7) and postnatal day 45 (PN45).

### 2.3. Grouping and maternal propofol anesthesia

Three different investigators were involved in this study: the first investigator randomised the pregnant mice into 4 groups and administered the study drugs to each group as the only investigator aware of the treatment groups. The second investigator performed the immunohistochemistry and the third investigator performed the behavioural tests.

Twenty pregnant mice were divided into the following four groups, using a computer- based random order generator (n = 5 per group, Fig 1A):

1. **Group C:** 1 ml of saline was administered intraperitoneally daily throughout pregnancy and an intralipid solution as vehicle was administered intraperitoneally at G14. The offspring of these pregnant mice were termed group C.
2. **Group D:** Intraperitoneal vit D of 5 µg kg^-1^ was administered daily throughout pregnancy [18] and an intralipid solution as vehicle was administered intraperitoneally at G14. The offspring of these pregnant mice were termed as group D.
3. **Group P:** 1 cc of saline was administered intraperitoneally daily throughout pregnancy, and 200 mg kg^-1^ of propofol was administered intraperitoneally at G14 [19]. The offspring of these pregnant mice were termed group P.
4. **Group PD:** Intraperitoneal vit D of 5 µg kg^-1^ was administered daily throughout pregnancy and 200 mg kg^-1^ of propofol was administered intraperitoneally at G14 [18,19]. The offspring of these pregnant mice were termed group PD.

Based on previous studies, the intraperitoneal vit D dose for pregnant mice was determined to be 5 µg kg^-1^ [18]. Vit D was administered via the intraperitoneal route at this dose daily at the same time throughout pregnancy. Olive oil was used as the solvent. In previous studies, 200 mg kg^-1^ of intraperitoneal propofol provided anaesthesia and immobilisation in the surgical plane. In addition, O_2_ and CO_2_ partial pressures and pH in the arterial blood of the animals were found to be normal [19]. In this study, animals were placed in a heating device to maintain their body temperature at ~37^o^C and breathed spontaneously during anaesthesia. Oxygen saturation was monitored by pulse oximetry. Throughout the experiments, there was no evidence of respiratory depression or decrease in oxygenation in the mice. Once the righting reflex was restored, the pregnant mice were safely returned to their cage.

### 2.4. Behavioral test of the offspring mice

To minimize interaction between offspring, one male offspring from each litter was randomly assigned to either the control or experimental groups. Individual identification was achieved by marking the tail with a tattoo pen. After the 3-day habituation period, male offspring were subjected to behavioural tests for assessment of emotional functioning on PN45.

After a three-day habituation period, male offspring underwent behavioral tests at one-day intervals to evaluate their emotional functions. The open field test (OFT) was conducted on PN45, and the elevated plus maze (EPM) test was performed on postnatal day 46 (PN46).

#### 2.4.1. Open field test (n = 8 per group).

Mice were brought into the laboratory and allowed to habituate for 5 minutes before the test. They were placed in the corner of an open field box (60 cm x 60 cm) and their behaviour observed for 5 minutes. The time spent at the center and outer zones, the distance travelled and frequency of entry into the centre were recorded and analysed using ETHOVISION XT (NOLDUS, version 4.1). The latency of the first entry into the centre and the frequency of standing up were scored manually. The temperature was set at 24°C and the light intensity at 100 lux.

#### 2.4.2. Elevated plus maze test (n = 8 per group).

The 40 cm-high maze has two open and two closed arms (30 x 5 cm²), which intersect. The open arms of the maze have a light setting of 165 lux, and the closed arms are surrounded by a 15 cm-high wall. Each mouse was placed in the centre of the maze with its face facing the open arm and allowed to explore for 5 minutes. The time spent open or closed, and frequency of head movement were recorded on video for 5 minutes and scored manually.

### 2.5. Immunohistochemical methods

Offspring interaction was minimized by randomly assigning one offspring from each litter to the control or experimental group. Individual identification was achieved by marking the tail with a tattoo pen. For the immunohistochemical (IH) analysis, tissue samples were obtained from the hippocampus and prefrontal cortex of six offspring from each group on PN7 and PN45. The tissues were fixed and embedded in formalin. Three sections from each of the six blocks obtained from each tissue were placed on slides and stained. IH staining methods specific for Bax (1/200; E-AB-13814, Elabscience, USA), Bcl-2 (1/200; E-AB-64075, Elabscience, USA), TNF-α (1/200; bs-10802R, Bioss, USA), IL-6 (1/200; bs-0782R, Bioss, USA), c-Fos (1/200; AF5354, Affinity, USA), Olig2 (1/400; DF8004, Affinity, USA), and BDNF (1/400; E-AB-18244, Elabscience, USA) were used. Sections were treated with primary antibodies at +4°C for a night then at room temperature for one hour. Biotin solution (ab93705, Abcam, USA) was dropped onto the sections, which were then incubated, passed through distilled water and PBS, left for fifteen minutes, and washed with tap water. Sections were blotted and avidin (ab93705, Abcam, USA) was added and incubated for 20 minutes at room temperature. AEC solution was dropped onto the sections. The sections were passed through distilled water and PBS. The sections were left for 10 minutes, then washed with tap water. Contrast staining with haematoxylin followed. The sections were examined under an Olympus BX53 light microscope. The reactivity of various markers was then assessed using a semiquantitative approach [20].

### 2.6. Immunohistochemical assessment

The H-score system was used to score 10 random areas using a 40 objective. The H-score = product of 4 staining intensities × percentage of positive cells. The degree of staining was scored as follows: 0 = no staining, 1 = weak, 2 = moderate, 3 = strong. The H-score value was calculated using the formula I (intensity) x PC (percentage of positive cells).

### 2.7. Electron microscopic methods

For EM analysis, tissue samples were taken from the hippocampus and prefrontal cortex of six mice in each group. Four blocks were obtained from each sample. Five different areas from each block were examined and photographed under an EM. Tissue samples were fixed in 5% glutaraldehyde in phosphate buffer (pH 7.2) for 4 hours and postfixed in 1% osmium tetroxide. They were then dehydrated, embedded in araldite and processed for electron microscopy [21]. The stained sections were examined using a JEOL-JEM 1400 transmission electron microscope (Japan).

### 2.8. Statistical methods

Data were expressed as mean ± standard deviation (SD). Differences in behavioral tests were considered significant at p < 0.05. Non-normally distributed and non-parametric data were analyzed using the Kruskal-Wallis test between more than two groups. The Mann-Whitney U test with a Bonferroni correction was used for pairwise comparisons of groups for situations found significant in these comparisons.

Numerical measurements of IH data were expressed as mean ± SD. Comparisons of two dependent, non-normally distributed numerical measurements were analyzed using the Wilcoxon Signed Rank test. The Shipiro-Wilk test for normality was applied as the sample size was less than 30. One-way analysis of variance (ANOVA) was used to compare numerical measurements of more than two groups. Scheffe tests were used for pairwise comparisons of groups depending on whether the variances within the groups were homogeneous. IBM SPSS Statistics Version 20.0 (IBM Corp.; Armonk, NY, USA) was used for statistical analysis of the data. The threshold for statistical significance was p < 0.05 in all tests.

The primary outcome of the study was to examine the effect of vitamin D on propofol-induced neuroinflammation and apoptosis. To evaluate the statistical power, the R-squared was calculated using the study data and according to these calculations, the power of the study was found to be 98.8%.

## 3. Results

### 3.1. Propofol has no effect on adulthood anxiety and locomotor activity

In the OFT, no significant difference was found between the groups for time spent in the centre and outer zones [H(3,32) = 2.18, p = 0.53; figure 1-B1, figure 1-B2], distance travelled [U(3,32) = 4.47, p = 0.21; figure 1-B3] and frequency of movement to the center [U(3,32) = 4.39, p = 0.22; figure 1-B4].

In the EPM, the time spent in the open arm [H(3,32) = 2.25, p = 0.52; figure 1-C1], the time spent in the closed arm [H(3,32) = 1.23, p = 0.74; figure 1-C2], the frequency of entering the open arm [H(3,32) = 2.90, p = 0. 40; figure 1-C3], the latency to enter the open arms [H(3,32) = 3.53, p = 0.31], the frequency of extending the head downwards from the open arm [H(3,32) = 6.00, p = 0.11] were not significantly different between the groups. These data suggest that intrauterine propofol exposure does not affect locomotor activity and anxiety-like behaviour in adulthood.

### 3.2. Vitamin D attenuates maternal propofol-induced neuroinflammation in the hippocampus and prefrontal cortex of offsprings

To investigate the effects of vit D on maternal propofol-induced neurotoxicity, we identified proteins involved in this process by immunohistochemistry. For immunohistochemical evaluation, hippocampus and prefrontal cortex tissues were collected from offspring at PN7 and PN45. Both male and female mice were used in the immunohistochemical studies, and there were no gender-based differences between the groups.

**Fig 2:**
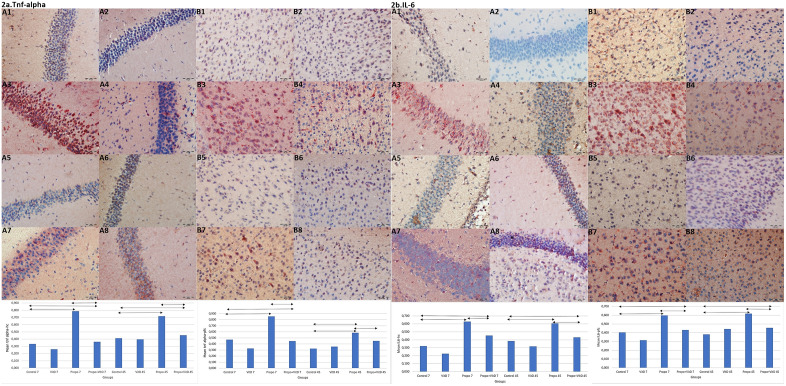
TNF-alpha and IL-6 IR in hc and pfc. a: TNF-α IR in hp and pfc. A1. Minimal IR in hp neurons, group C at PN7. A2. Weak positive IR in hc, group D at PN7. A3. High positive IR in hc, group P at PN7. A4. Weak positive IR in hc, group PD at PN7. A5. Minimal IR in hc, group C at PN45. A6. Minimal IR in hc, group D at PN45. A7. Highly positive IR in hc, group P at PN45. A8. Weak positive IR in hp, group PD at PN45. B1. Minimal IR in pfc, group C at PN7. B2. Weak positive IR in pfc, group D, PN7. B3. Strong positive IR in pfc, group P, PN7. B4. Weak positive IR in pfc, group PD, PN7. B5. Minimal IR in pfc, group C at PN45. B6. Minimal IR in pfc, group D at PN45. B7. Positive IR in pfc, group P at PN45. B8. Weak positive IR in pfc, group PD, at PN45. b: IL-6 IR in the hp and pfc. A1. Minimal IR in hc, group C at PN7. A2. Weak positive IR in hc, group D at PN7. A3. Positive IR in hp, group P at PN7. A4. Weakly positive IR in hp, group PD at PN7. A5. Minimal IR in hp, group C at PN45. A6. Minimal IR in hp, group D at PN45. A7. Decreased IR in hp, group P at PN45. A8. Weak positive IR in hp, group PD at PN45. B1. Minimal IR in pfc, group C at PN7. B2. Weak positive IR in pfc, group D at PN7. B3. Strong positive IR in pfc, group P at PN7. B4. Weak positive IR in pfc, group PD PN7. B5. Minimal IR in pfc, group C at PN45. B6. Minimal IR in pfc, the group D at PN45. B7. Strong IR in pfc, group P at PN45. B8. Weak positive IR in pfc, PD group at PN45. Scale Bar: 50 μm. IH scoring of IL-6 in hipocampus. 2a/ C1: IH scoring of IL-6 in hipocampus. 2a/ C2: IH scoring of IL-6 in prefrontal cortex. 2b/ C1. IH scoring of TNF-α in hipocampus. 2b/ C2: IH scoring of TNF-α in prefrontal cortex. Immunoreactivity: IR, Immunohistochemical: IH, PN7: Postnatal 7th day, PN45: Postnatal 45th day. Comparisons were analyzed using the Wilcoxon Signed Rank testand One-way variance analysis. p < 0.001‌‌.

In order to examine the mechanism by which vit D reduces propofol-induced neuroinflammation, two important inflammatory factors, IL-6 and TNF-α, were evaluated in the hippocampus and prefrontal cortex of offsprings. We found that intrauterine exposure to propofol led to a significant increase in the levels of IL-6 and TNF-α, while vit D treatment resulted in a reduction of these levels at PN7 and PN45 (**p < 0.001**, S1 Table, S2 Table, Fig 2). These results demonstrated that vit D might attenuate maternal propofol-induced neuroinflammation.

### 3.3. Vitamin D mitigates maternal propofol-induced apoptosis in the offsprings’ hippocampus and prefrontal cortex

**Fig 3:**
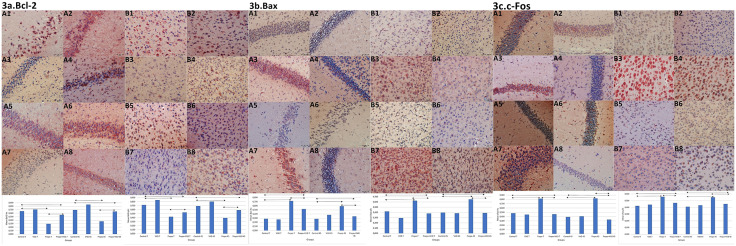
Bcl-2, Bax and c-Fos IR in hp and pfc. a: Bcl-2 IR in hp and pfc. A1. High positive IR in hp, group C at PN7. A2. Positive IR in hp, group D at PN7. A3. Minimal positive IR in hc, group Pat PN7. A4. Positive IR in hp, group PD at PN7. A5. Positive IR in hp, group C at PN45. A6. Highly positive IR in hp, group D at PN45. A7. Weakly positive IR in hp, group P at PN45. A8. Positive IR in hp, group PD ay PN45. B1. Strong positive IR in pfc, group C at PN7. B2. Positive IR in pfc, group D at PN7. B3. Weakly positive IR in pfc neurons, group P at PN7. B4. Positive IR in pfc, group PD at PN7. B5. Positive IR in pfc, group C at PN45. B6. Positive IR in pfc, group D at PN45. B7. Weakly positive IR in pfc, group P at PN45. B8. Positive IR in pfc, group PD at PN45. b: Bax IR in hp and pfc. A1. Minimal IR in hp, group C at PN7. A2. Weak positive IR in hp, group D at PN7. A3. Highly positive IR in hp, group P at PN7. A4. Weakly positive IR in hp, group PD at PN7. A5. Minimal IR in hp, group C at PN45. A6. Weakly IR in hp, group D at PN45. A7. Positive IR in hp, group P at PN45. A8. Weak positive IR in hp, group PD at PN45. B1. Minimal IR in pfc, group C at PN7. B2. Weak positive IR in pfc, group D at PN7. B3. Strong positive IR in pfc, group P at PN7. B4. Weak positive IR in pfc, PD group at PN7. B5. Minimal IR in pfc, group C at PN45. B6. Minimal IR in pfc, group D at PN45. B7. Highly positive IR in pfc, group P at PN45. B8. Positive IR in pfc, group PD at PN45. c: c-Fos IR in hp and pfc. A1. Minimal IR in hp, group C at PN7. A2. Weak positive IR in hp, group D at PN7. A3. Highly positive IR in hp, group P at PN7. A4. Weakly positive IR in hp neurons, group PD at PN7. A5. Minimal IR in hp, group C at PN45. A6. Weakly IR in hp neurons, group D at PN45. A7. Strong positive IR in hp, group P at PN45. A8. Weak positive IR in hp, group PD at PN45. B1. Minimal IR in pfc, group C at PN7. B2. Weak positive IR in pfc, group D at PN7. B3. Strong positive IR in pfc, group P at PN7. B4. Weak positive IR in pfc, group PD at PN7. B5. Minimal IR in pfc, group C at PN45. B6. Minimal IR in pfc, group D at PN45. B7. Highly positive IR in pfc, group P at PN45. B8. Positive IR in pfc, group PD at PN45. Scale Bar: 50 μm. 3a/ C1. IH scoring of Bcl-2 in hipocampus. 3a/ C2: IH scoring of Bcl-2 in pfc. 3b/ C1. IH scoring of Bax in hipocampus. 3b/ C2: IH scoring of Bax in prefrontal cortex. 3c/ C1. IH scoring of c-Fos in hipocampus. 3c/ C2. IH scoring of c-Fos in prefrontal cortex. IR: Immunoreactive, IH: Immunohistochemical, hp: hippocampus, pfc: prefrontal cortex, PN7: Postnatal 7th day, PN45: Postnatal 45th day. Comparisons were analyzed using the Wilcoxon Signed Rank test and One-way variance analysis. p < 0.001‌‌.

In the developing brain, Bax and Bcl-2 from the Bcl-2 protein family have been identified as the primary regulators of apoptosis. Bax acts pro-apoptotic and Bcl-2 acts anti-apoptotic in the intrinsic mitochondrial pathway [22]. c-Fos has been linked to apoptosis in response to antiproliferative conditions and cellular damage [23]. Our results showed that maternal propofol exposure significantly increased Bax and c-Fos immunoreactivity and decreased Bcl-2 immunoreactivity in offspring neurons of group P compared to other groups at PN7 and PN45. Notably, maternal vitamin D treatment resulted in increased Bcl-2 expression and decreased Bax and c-Fos expression in neurons exposed to propofol **(p < 0.001**, S1, S2 Tables, Fig 3). These findings suggest that maternal propofol-induced significant apoptosis in the developing brain can be prevented by vit D.

**Fig 4:**
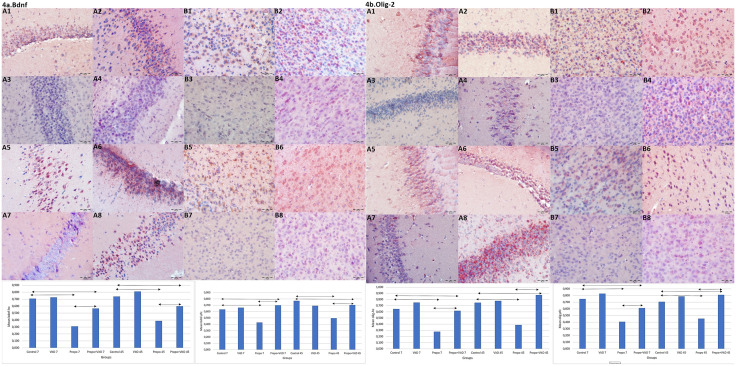
Bdnf and Olig-2 IR in hp and pfc. a: BDNF IR in hp and pfc. A1. Positive IR in hp, group C at PN7. A2. Strong positive IR in hp, group D at PN7. A3. Minimal positive IR in hp, group P at PN7. A4. Positive IR in hp, group PD at PN7. A5. Higly positive IR in hp, group C at PN45. A6. Highly positive IR in hp, group D at PN45. A7. Weakly positive IR in hp, group P at PN45. A8. Positive IR in hp, group PD at PN45. B1. Positive IR in pfc, group C at PN7. B2. Positive IR in pfc, group D at PN7. B3. Weakly positive IR in pfc, group P at PN7. B4. Positive IR in pfc, group PD at PN7. B5. Positive IR in pfc, group C at PN45. B6. Positive IR in pfc, group D at PN45. B7. Minimal positive IR in pfc, group P at PN45. B8. Positive IR in pfc, group PD at PN45. b: Olig2 IR in hp and pfc. A1. Highly positive IR in hp, group C at PN7. A2. Strong positive IR in hp, group D at PN7. A3. Weakly positive IR in hp, group P at PN7. A4. Positive IR in hp, group PD at PN7. A5. Strong positive IR in hp, group C at PN45. A6. Highly positive IR in hp, group D at PN45. A7. Weak positive IR in hp, group P at PN45. A8. Positive IR in hp, group PD at PN45. B1. Positive IR in pfc, group C at PN7. B2. Positive IR in pfc, group D at PN7. B3. Weak positive IR in pfc, group P at PN7. B4. Positive IR in pfc, group PD at PN7. B5. Positive IR in pfc, group C at PN45. B6. Positive IR in pfc, group D at PN45. B7. Minimal positive IR in pfc, group P at PN45. B8. Positive IR in pfc, group PD at PN45. Scale Bar: 50 μm. 4a/ C1. IH scoring of BDNF in hp. 4a/ C2: IH scoring of BDNF in pfc. 4b/ C1. IH scoring of Olig2 in hipocampus. 4b/ C2: IH scoring of Olig2 in prefrontal cortex. IR: Immunoreactivity, IH: Immunohistochemical, hp: hippocampus, pfc: prefrontal cortex, PN7: Postnatal 7th day, PN45: Postnatal 45th day. Comparisons were analyzed using the Wilcoxon Signed Rank test and One-way variance analysis. p < 0.001‌‌.

Brain-derived neurotrophic factor (BDNF) is crucial for neuronal survival, growth and development, and has been shown to increase dendritic spine size and density [24]. Levels of BDNF were found to be significantly increased in group D in comparison to the other groups at PN7 and PN45 (**p < 0.001**, Fig 4a, S1, S2 Tables). Furthermore, neuronal expression of BDNF in group PD significantly increased in comparison to group P at PN7 and PN45 (p < 0.001, Fig 4a, S1, S2 Tables). These results indicate that vit D improves BDNF levels and can prevent propofol-induced neurotoxicity.

**Fig 5:**
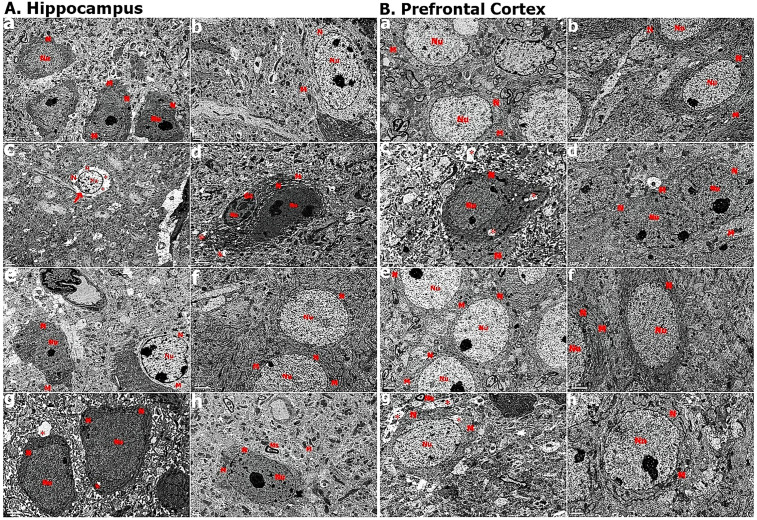
EM examinations of hippocampus and prefrontal cortex tissue sections. 5/ A: EM examination of hippocampus tissue sections. 5/ A-a. Group C at PN7. 5/ A-b. Group D at PN7. 5/ A-c. Group P at PN7 (Red arrow: neural degeneration). 5/A- d. Group PD at PN7. 5/ A-e. Group C at PN45. 5/ A-f. Group D at PN45. 5/ A-g. Group P at PN45. 5/A- h. Group PD at PN45 5/ B: EM examination of prefrontal cortex tissue sections. 5/ B-a. Group C at PN7. 5/ B-b. Group D at PN7. 5/ B-c. Group P at PN7. 5/ B- d. Group PD at PN7. 5/ B-e. Group C at PN45. 5/ A-f. Group D at PN45. 5/ B-g. Group P at PN45. 5/ B- h. Group PD at PN45. Scale bar: 2 μm. N: Neuron, Nu: Nucleus, M: Mitochondria. Gc: Glial cell. PN7: Postnatal 7th day, PN45: Postnatal 45th day. Red *: neural edematous degeneration, Red arrow: heterochromatin increase in the nucleus‌‌.

Our study found that vit D reduced the levels of apoptotic proteins caused by intrauterine propofol. This was confirmed by EM examination of the hippocampus and prefrontal cortex tissues. EM of group P revealed increased electron density, vacuolisation, intrastoplasmic oedematous changes and myelin sheath degeneration in neurons at PN7 and PN45. The PD group exhibited normal neuron and glia cell structures (Fig 5).

### 3.4. Vit D increases the expression of Olig2 suppressed by maternal propofol anesthesia in the offsprings’ brain

Olig2 is a transcriptional protein that plays an important role in modulating the specification and differentiation of oligodendrocytes (OL) [25]. In this study, Olig2 expression was significantly higher in group D than in the other groups (Fig 4b, S1, S2 Tables, p < 0.001). Furthermore, Olig2 expression in Group PD was significantly higher than in Group P (Fig 4b). Our results indicated that intrauterine propofol exposure exerts an inhibitory effect on myelination by decreasing Olig2 expression, and maternal vit D treatment may alleviate this inhibition in offspring.

## 4. Dıscussıon

During the midgestational and early postnatal period, neurons are highly susceptible to the neurotoxic effects of general anaesthetics [26]. Studies have shown that exposure to propofol may be associated with neurotoxicity in the developing brain [11,12]. This study investigated the protective effects of vit D on maternal propofol-induced neurotoxicity. Similar to previous studies, we found that maternal exposure to propofol caused neuroinflammation and apoptosis in the offspring. Importantly, treatment with vit D during pregnancy reduced this neurotoxicity.

Factors such as calcium accumulation, mitochondrial fusion, neuroinflammation and impaired neurotrophin expression are thought to be the mechanisms underlying propofol-induced neurotoxicity [27,28]. Microglia are small immune cells distributed throughout the brain tissue that react quickly to changes in the brain’s microenvironment [29]. TNF-α, a proinflammatory cytokine, has been demonstrated to activate microglia in various brain regions and to increase the release of other cytokines [27]. Animal experiments have demonstrated that propofol exposure can lead to increased pro-inflammatory factors, such as IL-6 and TNF-α, neural injury, and apoptosis in rat hippocampal tissues [30]. Previous studies have generally focused on the neonatal period, but our study investigated the effects of propofol exposure in the prenatal period on neuroinflammation. The hippocampus and prefrontal cortex are anatomical areas particularly sensitive to neurotoxicity caused by anaesthetic agents [22,30], and the inflammatory response in these cerebral areas in offspring exposed to propofol in the second trimester of pregnancy was therefore examined. It was found that TNF-α and IL-6 levels were significantly increased compared to the control group in both PN7 and PN45.

Li et al. [31] observed significant microglial activation six hours after foetal exposure to propofol. They determined that this activation was most prominent in the cortex and thalamus, and was associated with neuroapoptosis. It is well-established that activated microglia may cause age-related neurodegeneration by releasing a variety of proinflammatory and possibly neurotoxic factors [29,32]. In the present study we found that TNF-α and IL-6 levels were significantly higher than the control group in PN45. This finding may be attributed to microglial activation potentially induced by intrauterine propofol exposure; however, further studies are required to confirm this association.

Vitamin D has been demonstrated to function as an immunomodulator, thereby regulating the expression of proinflammatory enzymes and cytokine genes [33,34]. It has been demonstrated that pretreating aged mice with vit D can alleviate sevoflurane-induced neuroinflammation and reduce increased inflammatory mediators in these animals [35]. Another study on ageing rats found that vit D pre-treatment significantly improved memory and cognitive function impaired by surgery, by inhibiting neuroinflammation [36]. Ali et al. [37] reported that vit D reduced neuroinflammation, synaptic dysfunction and memory impairment by suppressing TNF-α and IL-1β in animal models of ageing and neurodegenerative diseases. In the present study, propofol-induced IL-6 and TNF-α expressions significantly reduced in the offspring of mice that received vit D treatment throughout pregnancy compared to the offspring of untreated mice. Our results suggested that maternal propofol-induced neuroinflammation can be prevented with vit D in the offsprings.

The hippocampus, a brain region involved in memory and learning, is particularly vulnerable to propofol-induced apoptosis due to its effect on mitochondria [22,38,39]. The phenomenon of propofol-induced neurodegeneration involves intrinsic (mitochondrial) and extrinsic (receptor-mediated) apoptotic pathways. These are mediated by the Bcl-2 protein family and TNF-α [22]. It has also been demonstrated that c-Fos-induced apoptosis is inhibited by Bcl-2 [23]. This study found lower Bcl-2 and higher Bax, c-Fos, TNF-α and IL6 levels in the hippocampal and prefrontal cortex tissues of offspring exposed to prenatal propofol. EM showed increased electron density in neurons, vacuolation and myelin sheath degeneration. These findings provide a robust scientific rationale for the immunohistochemical results of our study.

Vitamin D has been identified as a neuroactive steroid with neuroprotective and anti-apoptotic functions in the CNS [40, 41]. Guo et al. [41] reported that vit D administration in rats with global cerebral ischemia reduced apoptosis by reversing the ischemia-induced increase in Bax and decrease in Bcl-2 in the hippocampus. Consistent with Guo et al., the present study has provided evidence that treatment of pregnant mice with vitamin D resulted in a reduction in the expression of Bax and TNF-α, and an increase in the expression of Bcl2, in the offspring exposed to intrauterine propofol.

For immature neurons to function properly, they need a more suitable environment containing neurotrophic growth factors to form and support synaptic structures. One of these factors, BDNF plays a key role in the growth, differentiation, survival and plasticity of neurons [24]. Intraperitoneal administration of propofol to pregnant rats in the first trimester significantly reduced BDNF expression in the offspring [42]. Vit D has been shown to exert neuroprotective effects by modulating the production of neurotrophins such as nerve growth factor (NGF) and BDNF in the brain [15,43]. It has been shown that vitamin D acts as a potent differentiation agent, inducing NGF in rat hippocampal cultures [43]. Şahin et al. [44] induced hippocampal apoptosis in rats treated with prophylactic vit D. They reported that vit D prevented apoptosis by increasing BDNF and decreasing Bax, caspase-3 and c-Fos. In the present study, we observed that levels of BDNF were significantly increased in the offspring of mice treated with vit D in comparison with the offspring of untreated mice. Our results suggested that vit D may have an important role in neuroprotection against the neurotoxic effects of propofol through BDNF.

Myelin sheath, a structural component of axonal transmission, is produced by OLs. OLs express the VDR, and vitamin D deficiency results in decreased differentiation of these cells and subsequent demyelination [45]. Animal and laboratory studies have demonstrated that propofol anaesthesia can induce OL apoptosis [12,46]. Olig2 is a regulator OL differentiation [25]. In the present study, treatment of pregnant mice with vit D throughout gestation resulted in elevated Olig2 levels their offspring, in cases where the mice had been exposed to propofol.

γ-Aminobutyric acid (GABA) is a trophic factor in the developing brain. Changes in receptor structure, distribution, and brain neurotrophin concentration after fetal exposure to GABA receptor modulators such as benzodiazepines and ethanol may play a role in anxiety-related behaviours in adulthood [47]. In addition, propofol is able to induce neuronal apoptosis in young rats and cause brain dysfunction by activating the GABA receptor [48]. Zhou et al. [49] reported that neonatal exposure to propofol led to motor learning disorders, and anxiety-like behaviours in adult mice. Two other studies conducted on newborn rats showed that propofol exposure caused minor behavioural changes in the OFT but did not affect the learning and memory function of adolescent animals [50,51]. Li et al. [31] found that offspring exposed to propofol on the G20 did not differ from the control group in spontaneous locomotor activity tests on the PN35. However, functional differences in radial maze performance were only evident on days one, two and three of the five-day behavioural test. The current study investigated the adulthood locomotor activity and anxiety levels of mice exposed to prenatal propofol, using OFT and EPM. Despite the detection of significant differences in immunohistochemical examinations, no differences in anxiety-like behaviours were identified between the groups. The different results observed in behavioural tests, including in our own study, make it challenging to determine the behavioural consequences of propofol-induced neurodegeneration. Although propofol exerts its anaesthetic effect through the GABA receptor, the GABA receptor is not the only factor in propofol-induced neuro-apoptosis in the developing brain [52]. Furthermore the temporal parameters of propofol administration have the potential to yield divergent behavioural outcomes. Further comprehensive studies on this subject are therefore necessary.

Our study has several important limitations. Despite the absence of significant respiratory or oxygenation complications during the study, other physiological effects that may occur during pregnancy (e.g., stress reaction, inflammation, haemodynamic changes) were not systematically monitored. This is a primary limitation in interpreting the results. Predictable confounding variables, such as environmental factors, nutritional conditions and birth weight, were distributed evenly across the groups and therefore did not affect the results. Propofol (200 mg/kg) was used to induce general anaesthesia in pregnant mice. Different doses, or repeated doses, could have yielded different results, but this was not analysed. Since propofol and vit D are administered intravenously in clinical practice, the pharmacokinetics of the agents used may have been affected by their intraperitoneal administration. Finally, since the pregnant mice did not undergo surgery, the contribution of the surgical stress response to propofol toxicity in the offspring could not be investigated. Consequently, the results should be interpreted in light of these methodological limitations.

### 4.1. Conclusion

Our data revealed that the prophylactic administration of vit D during pregnancy could prevent neuroapoptosis and neuroinflammation in the offspring of mice exposed to propofol. Consequently, we suggest that intrauterine propofol-induced neurotoxicity in infants could be mitigated by maintaining adequate levels of vit D in maternal plasma. However, further research is necessary to investigate the clinical relevance of these findings, taking into account the limitations of the present study, and to explore the potential of vitamin D as an adjunctive treatment to enhance paediatric patient care in anaesthesia practice.

## Supporting information

S1 Table Results of the comparison of all measurements on the 7th postnatal day between the groups. (DOCX)

S2 Table Results of the comparison of all measurements on the 45th postnatal day between the groups. (DOCX)

S1 File Immunohistochemical assessment data. (XLSX)

S2 File Elevated plus maze data. (XLSX)

S3 File Open field test data. (XLSX)
