# Supplementary material for: Vitamin D alleviates neurotoxicity induced by propofol anaesthesia in the offspring of mice
Source: PLoS One. 2026 May 22;21(5):e0349784. doi: 10.1371/journal.pone.0349784 (PMC13196955; doi:10.1371/journal.pone.0349784)
Supplement: S1 Table — (DOCX) [file pone.0349784.s001.docx]

**S1 Table:**Results of the comparison of all measurements on the 7th postnatal day between the groups

| **Variables** | **Group C7** | **Group D7** | **Group P7** | **Group PD7** | **p*** |
| --- | --- | --- | --- | --- | --- |
| **Bax-hc** | 0.28±0.04  (0.24-0.33) | 0.27±0.02  (0.24-0.29) | 0.72±0.02  (0.69-0.74) | 0.52±0.02  (0.5-0.54) | < 0.001^b,c,d,e,f^ |
| **Bax-pfc** | 0.42±0.04  (0.38-0.46) | 0.29±0.02  (0.27-0.32) | 0.62±0.04  (0.58-0.67) | 0.38±0.02  (0.36-0.4) | < 0.001^a,b,d,f^ |
| **Bcl2-hc** | 0.66±0.05  (0.61-0.71) | 0.71±0.03  (0.68-0.74) | 0.3±0.03  (0.27-0.33) | 0.55±0.02  (0.53-0.57) | < 0.001^b,c,d,e,f^ |
| **Bcl2-pfc** | 0.72±0.02  (0.7-0.74) | 0.84±0.03  (0.81-0.88) | 0.42±0.02  (0.4-0.45) | 0.53±0.03  (0.49-0.57) | < 0.001^b,c,d,e,f^ |
| **Tnfα-hc** | 0.33±0.02  (0.32-0.35) | 0.26±0.01  (0.25-0.27) | 0.79±0.02  (0.76-0.81) | 0.36±0.02  (0.34-0.39) | < 0.001^a,b,d,e,f^ |
| **Tnfα-pfc** | 0.47±0.02  (0.45-0.5) | 0.33±0.03  (0.3-0.35) | 0.86±0.03  (0.82-0.89) | 0.45±0.03  (0.42-0.48) | < 0.001^b,d,e,f^ |
| **IL6-hc** | 0.32±0.01  (0.31-0.33) | 0.23±0.02  (0.2-0.25) | 0.63±0.03  (0.6-0.66) | 0.45±0.02  (0.43-0.48) | < 0.001^a,b,c,d,e,f^ |
| **IL6-pfc** | 0.4±0.02  (0.38-0.43) | 0.32±0.02  (0.29-0.34) | 0.6±0.03  (0.57-0.62) | 0.43±0.02  (0.41-0.45) | < 0.001^a,b,d,e,f^ |
| **cFos-hc** | 0.48±0.03  (0.45-0.51) | 0.45±0.02  (0.42-0.47) | 0.82±0.03  (0.79-0.84) | 0.46±0.02  (0.43-0.48) | < 0.001^b,d,f^ |
| **cFos-pfc** | 0.42±0.03  (0.39-0.45) | 0.44±0.03  (0.41-0.48) | 0.56±0.03  (0.53-0.59) | 0.47±0.02  (0.45-0.49) | < 0.001^a,b,d,f^ |
| **Olig2-hc** | 0.65±0.03  (0.62-0.67) | 0.75±0.02  (0.73-0.77) | 0.28±0.02  (0.26-0.3) | 0.62±0.05  (0.56-0.67) | < 0.001^a,b,d,e,f^ |
| **Olig2pfc** | 0.75±0.03  (0.72-0.78) | 0.83±0.02  (0.81-0.85) | 0.41±0.02  (0.39-0.42) | 0.61±0.02  (0.59-0.63) | < 0.001^a,b,c,d,e,f^ |
| **Bdnf-hc** | 0.71±0.02  (0.69-0.73) | 0.73±0.02  (0.7-0.75) | 0.31±0.02  (0.29-0.33) | 0.57±0.02  (0.54-0.59) | < 0.001^b,c,d,e,f^ |
| **Bdnf-pfc** | 0.63±0.02  (0.61-0.65) | 0.66±0.02  (0.65-0.68) | 0.43±0.03  (0.4-0.46) | 0.7±0.02  (0.68-0.72) | < 0.001^b,c,d,f^ |

*The mean and standard deviation and 95% confidence interval were presented. Tukey test for pairwise comparisons: ^a^p < 0.05 for Group C vs Group D, ^b^p < 0.05 for Group C vs Group P, ^c^p < 0.05 for Group C vs Group PD, ^d^p < 0.05 for Group D vs Group P, ^e^p < 0.05 for Group D vs Gruop PD, ^f^p < 0.05 for Group P vs Group PD. * p value of variance analysis (ANOVA) Hp: hippocampus, pfc: prefrontal cortex.*
