# Supplementary material for: Vitamin D alleviates neurotoxicity induced by propofol anaesthesia in the offspring of mice
Source: PLoS One. 2026 May 22;21(5):e0349784. doi: 10.1371/journal.pone.0349784 (PMC13196955; doi:10.1371/journal.pone.0349784)
Supplement: S2 Table — (DOCX) [file pone.0349784.s002.docx]

**S2 Table:** Results of the comparison of all measurements on the 45th postnatal day between the groups

| **Variables** | **Group C45** | **Group D45** | **Group P45** | **Group PD45** | **p*** |
| --- | --- | --- | --- | --- | --- |
| **Bax-hc** | 0.28±0.01  (0.27-0.3) | 0.38±0.03  (0.35-0.41) | 0.59±0.02  (0.57-0.61) | 0.35±0.02  (0.32-0.37) | < 0.001^a,b,c,d,f^ |
| **Bax-pfc** | 0.4±0.02  (0.38-0.42) | 0.39±0.02  (0.36-0.41) | 0.65±0.02  (0.63-0.67) | 0.39±0.02  (0.37-0.41) | < 0.001^b,d,f^ |
| **Bcl2-hc** | 0.7±0.02  (0.68-0.71) | 0.85±0.03  (0.82-0.88) | 0.37±0.02  (0.35-0.39) | 0.65±0.03  (0.61-0.68) | < 0.001^a,b,d,e,f^ |
| **Bcl2-pfc** | 0.69±0.02  (0.68-0.71) | 0.8±0.02  (0.78-0.83) | 0.39±0.02  (0.38-0.41) | 0.59±0.02  (0.57-0.61) | < 0.001^a,b,c,d,e,f^ |
| **Tnfα-hc** | 0.41±0.03  (0.38-0.44) | 0.4±0.02  (0.38-0.41) | 0.72±0.03  (0.69-0.75) | 0.46±0.04  (0.41-0.5) | < 0.001^b,d,f^ |
| **Tnfα-pfc** | 0.32±0.03  (0.3-0.35) | 0.36±0.03  (0.33-0.38) | 0.59±0.02  (0.56-0.61) | 0.45±0.02  (0.43-0.47) | < 0.001^b,c,d,e,f^ |
| **IL6-hc** | 0.38±0.03  (0.36-0.41) | 0.32±0.03  (0.29-0.35) | 0.61±0.02  (0.58-0.63) | 0.43±0.03  (0.41-0.46) | < 0.001^b,d,e,f^ |
| **IL6-pfc** | 0.38±0.02  (0.36-0.4) | 0.44±0.03  (0.41-0.47) | 0.62±0.03  (0.59-0.64) | 0.46±0.02  (0.43-0.48) | < 0.001^b,c,d,f^ |
| **cFos-hc** | 0.39±0.01  (0.38-0.41) | 0.41±0.02  (0.39-0.43) | 0.82±0.03  (0.8-0.85) | 0.33±0.02  (0.32-0.35) | < 0.001^b,d,e,f^ |
| **cFos-pfc** | 0.41±0.02  (0.39-0.43) | 0.43±0.02  (0.41-0.46) | 0.55±0.02  (0.53-0.58) | 0.45±0.02  (0.43-0.48) | < 0.001^b,d,f^ |
| **Olig2-hc** | 0.75±0.03  (0.72-0.78) | 0.78±0.02  (0.76-0.8) | 0.39±0.03  (0.36-0.41) | 0.87±0.02  (0.85-0.9) | < 0.001^b,c,d,e,f^ |
| **Olig2pfc** | 0.71±0.02  (0.69-0.72) | 0.79±0.02  (0.77-0.8) | 0.45±0.02  (0.44-0.47) | 0.81±0.02  (0.79-0.83) | < 0.001^a,b,c,d,f^ |
| **Bdnf-hc** | 0.74±0.02  (0.72-0.76) | 0.81±0.02  (0.79-0.83) | 0.39±0.02  (0.36-0.41) | 0.6±0.02  (0.58-0.62) | < 0.001^a,b,c,d,e,f^ |
| **Bdnf-pfc** | 0.77±0.03  (0.74-0.8) | 0.69±0.02  (0.67-0.72) | 0.5±0.03  (0.46-0.53) | 0.7±0.04  (0.66-0.74) | < 0.001^b,d,f^ |

*The mean and standard deviation and 95% confidence interval were presented. Tukey test for pairwise comparisons: ^a^p < 0.05 for Group C vs Group D, ^b^p < 0.05 for Group C vs Group P, ^c^p < 0.05 for Group C vs Group PD, ^d^p < 0.05 for Group D vs Group P, ^e^p < 0.05 for Group D vs Gruop PD, ^f^p < 0.05 for Group P vs Group PD. * p value of variance analysis (ANOVA) Hp: hippocampus, pfc: prefrontal cortex.*
